# Immunomodulatory Effect of Adipose-Derived Stem Cells: The Cutting Edge of Clinical Application

**DOI:** 10.3389/fcell.2020.00236

**Published:** 2020-04-17

**Authors:** Simona Ceccarelli, Paola Pontecorvi, Eleni Anastasiadou, Claudio Napoli, Cinzia Marchese

**Affiliations:** ^1^Department of Experimental Medicine, Sapienza University of Rome, Rome, Italy; ^2^Clinical Department of Internal Medicine and Specialistics, Department of Advanced Clinical and Surgical Sciences, Università della Campania “Luigi Vanvitelli”, Naples, Italy; ^3^IRCCS SDN, Naples, Italy

**Keywords:** adipose-derived stem cells, immunomodulation, microenvironment, cytokines, clinical application

## Abstract

Adipose-derived stem cells (ASCs) represent a promising tool for soft tissue engineering as well as for clinical treatment of inflammatory and autoimmune pathologies. The well-characterized multi-differentiation potential and self-renewal properties of ASCs are coupled with their immunomodulatory ability in providing therapeutic efficacy. Yet, their impact in immune or inflammatory disorders might rely both on cell contact-dependent mechanisms and paracrine effects, resulting in the release of various soluble factors that regulate immune cells functions. Despite the widespread use of ASCs in clinical trials addressing several pathologies, the pathophysiological mechanisms at the basis of their clinical use have been not yet fully investigated. In particular, a thorough analysis of ASC immunomodulatory potential is mandatory. Here we explore such molecular mechanisms involved in ASC immunomodulatory properties, emphasizing the relevance of the milieu composition. We review the potential clinical use of ASC secretome as a mediator for immunomodulation, with a focus on *in vitro* and *in vivo* environmental conditions affecting clinical outcome. We describe some potential strategies for optimization of ASCs immunomodulatory capacity in clinical settings, which act either on adult stem cells gene expression and local microenvironment. Finally, we discuss the limitations of both allogeneic and autologous ASC use, highlighting the issues to be fixed in order to significantly improve the efficacy of ASC-based cell therapy.

## Introduction

Adipose-derived mesenchymal stem cells (ASCs) represent a population of self-renewing multipotent adult cells in the vascular stroma of adipose tissues, playing important roles in development, post-natal growth, maintenance of tissue homeostasis and tissue repair and regeneration (Shingyochi et al., 2015; Dai et al., 2016). Significant amount of ASCs can be readily accessible from subcutaneous liposuction and, when appropriately stimulated, they can further differentiate into several cell-like types, including adipocytes, osteocytes, neural cells, vascular endothelial cells, cardiomyocytes, pancreatic cells, and hepatocytes (Zuk et al., 2001, 2002; Planat-Bénard et al., 2004; Aurich et al., 2009; Dave et al., 2014; Sommese et al., 2017). To date, mechanisms responsible for ASC therapeutic efficacy have been only partially investigated. The common belief is that their action relies on three major events: (1) multi-differentiation potential, (2) self-renewal properties and (3) immunomodulatory capacity. In particular, the first could account for their impact on tissue engineering applications, due to the ability to differentiate into tissue-specific cells and into endothelial cells, thus stimulating both tissue regeneration and arteriogenesis. Indeed, several researchers have analyzed the efficacy of ASC-based therapy in restoring cardiac function in ischemic heart diseases (IHD) (Valina et al., 2007; Naaijkens et al., 2014), providing evidence of an extremely low rate of survival and cardiac retention of ASCs after transplantation. Such indications suggest that ASC therapeutic efficacy in IHD should be ascribed mainly to their paracrine effects rather than the direct differentiation into cardiovascular lineage cells (Yang et al., 2013). So, evaluation of ASC therapeutic potential should take into account both cell retention and survival after transplantation and production of active paracrine factors (Li et al., 2019).

The proliferative potential could represent a key element to consolidate the results obtained by ASC injection, leading to the generation of a relatively stable clone of self-renewing cells within the target tissue. Regarding the immunomodulatory capacity, it has been demonstrated that ASCs, as well as BMSCs, have a hypoimmunogenic phenotype, since they lack the major MHC class II molecules and express only low levels of MHC class I, this allowing them to evade immune recognition (Puissant et al., 2005; McIntosh et al., 2006). Furthermore, ASCs can act as modulators of the host response, showing a greater *in vitro* immunomodulatory ability than BMSCs derived from age-matched donors (Melief et al., 2013), since they are able to partially suppress lymphocytes proliferation, as well as to inhibit differentiation of monocyte-derived immature dendritic cells and NK cell cytotoxic activity (Russell et al., 2016; Valencia et al., 2016). Such effects are likely to depend on both cell contact-dependent mechanisms and paracrine effects through the production of cytokines and various soluble factors that regulate immune cells functions (Sotiropoulou et al., 2006), improve the microenvironment for tissue healing (Burlacu et al., 2013) and exert strong immunosuppressive effects by decreasing inflammatory cytokine production (Zhao et al., 2010). Indeed, higher immunomodulatory potential of ASCs is also related to higher levels of cytokine production (Melief et al., 2013).

These findings contributed to make ASCs a viable option in regenerative medicine and a powerful tool in cell-based therapy for restoring damaged tissues and decreasing inflammatory/immune response, opening the way to their application in the treatment of a wide panel of pathologies, including inflammatory and autoimmune diseases (De Miguel et al., 2012; Scuderi et al., 2013; Onesti et al., 2016). In preclinical studies, ASCs have been successfully used to reduce chronic disability in ischemic stroke in rats (Gutiérrez-Fernández et al., 2013; Oh et al., 2015; Chen et al., 2016), to delay onset and slow disease progression in murine and rat models of multiple sclerosis (Yousefi et al., 2013; Semon et al., 2014; Bowles et al., 2017) and to limit structural changes in the lung parenchyma by reducing inflammation and neutrophils number in the airways in chronic obstructive pulmonary disease in mice and guinea pig models (Ghorbani et al., 2014; Hong et al., 2016). Preclinical studies on ASCs, performed in swine and rodent models, also showed promising results across a wide range of cardiovascular therapeutic applications (Hashemi et al., 2008; Cai et al., 2009; Madonna et al., 2009; Bai et al., 2010; Grimaldi et al., 2013; Sommese et al., 2017), due to both stimulation of angiogenesis and potent anti-inflammatory paracrine effect eventually favoring the cardiac healing process (Gnecchi et al., 2005). ASC-based cellular therapy has been further considered for the treatment of neurodegenerative diseases, including mouse models of Alzheimer’s disease or Parkinson’s disease and amyotrophic lateral sclerosis (ALS) patients (McCoy et al., 2008; Yan et al., 2014; Fontanilla et al., 2015; Staff et al., 2016), as well as, in humans, for immunological disorders, such as graft versus host disease (GvHD) (Yañez et al., 2006; Fang et al., 2007; Tholpady et al., 2009) and autoimmune pathologies, such as type I diabetes mellitus (Vanikar et al., 2010; Lin et al., 2015), systemic sclerosis (Scuderi et al., 2013), rheumatoid arthritis (El-Jawhari et al., 2014; Ueyama et al., 2020) and systemic lupus erythematosus (SLE) (Liang et al., 2010; Park et al., 2015). A consistent number of clinical trials using ASCs are ongoing for the treatment of some of these disorders^1^, even though only some complete clinical results are now available, but the greatest number of human studies are in patients with osteoarthritis and inflammatory bowel disease (IBD) (González et al., 2009a; Sovrea et al., 2019). In particular, multiple Phase I clinical trials assessed the efficacy of intra articular injection of ASCs in improving pain, function and mobility of affected joints, with no major adverse effects (Jo et al., 2014; Pers et al., 2016; Yokota et al., 2017). As for IBD, in a phase III study, ASCs have been shown to be effective and safe for the treatment of complex perianal fistulas in Crohn’s disease patients who did not respond to conventional and/or biological treatments (Panés et al., 2016). The most relevant studies on ASCs-based treatment in different pathologies are summarized in Table 1.

**TABLE 1:** The most relevant studies on ASCs-based treatment in different pathologies are highlighted, indicating the type of treatment and the cellular and molecular mechanisms involved in ASCs or ASC secretome effect *in vivo*.

**Pathology**	** ASCs-based treatment**	**Cellular and molecular mechanisms**	**References**
Acute Myocardial Infarction	ASCs conditioned medium ASCs exosomes	Pro-survival and anti-apoptotic effects on cardiomyocytes; anti-inflammatory and pro-angiogenic potential (VEGF, HGF, PGF, PGE-2, FGF-2, TGF-β, IL-10, IDO, NO, Ang-1 and Ang-2, IGF-1, miR-31, miR-126 and miR-301a); Inhibition of fibrosis and cardiac remodeling (VEGF, HGF, MCP-1, TIMP-1 and TIMP-4)	Valina et al., 2007; Hashemi et al., 2008; Cai et al., 2009; Bai et al., 2010; Grimaldi et al., 2013; Naaijkens et al., 2014; Sommese et al., 2017

Cardiovascular diseases (e.g. acute ischemic stroke)	ASCs (intra-arterial and intravenous transplantation) ASCs exosomes	Attenuated inflammation and enhanced endogenous neurogenesis; reduction in cell death, increase in cellular proliferation, neurogenesis, oligodendrogenesis, synaptogenesis and angiogenesis markers; suppression of inflammation, generation of ROS and oxidative stress	Madonna et al., 2009; Gutiérrez-Fernández et al., 2013; Oh et al., 2015; Chen et al., 2016

Chronic Obstructive Pulmonary Disease	ASCs (intra-tracheal and intravenous delivery)	Reduction of oxidative damage; restored imbalance of protease/anti-protease ratio, anti-apoptotic activity and increased production of growth factors (HGF, FGF-2, VEGF): protection from lung damage	Ghorbani et al., 2014; Hong et al., 2016

Multiple Sclerosis	ASCs (intraperitoneal/intravenous injection) ASCs conditioned medium	Pathological features diminished, suppression of IFNγ and IL-12 in the sera of EAE mice; down-regulation of splenocytes proliferation and IL-17 secretion, alleviation of clinical scores severity; increased production of TGFβ, IL-4, IL-10, NO, and IDO by splenocytes	Yousefi et al., 2013; Semon et al., 2014; Bowles et al., 2017

Parkinson’s disease	ASCs (intra-striatal injection)	Production of growth factors known to promote dopaminergic survival and neuroprotection at the lesion site	McCoy et al., 2008

Alzheimer’s disease	ASCs (intra-hippocampal injection)	Reduced oxidative stress, alleviated cognitive impairment and enhanced neurogenesis	Yan et al., 2014

Amyotrophic Lateral Sclerosis	ASCs conditioned medium ASCs (intra-thecal delivery)	NGF-mediated neuroprotection: high motor neuron counts, less activation of microglia and astrocytes, reduced levels of phosphorylated p38 (inflammation and neural death) in the spinal cord; mild temporary subjective clinical improvements (phase I clinical trial)	Fontanilla et al., 2015; Staff et al., 2016

Systemic Sclerosis	ASCs (intra-dermal injection in hyaluronic acid solution)	Improvement in tightening of the skin due to anti-inflammatory soluble factors secretion and expansion of regulatory T-cells	Scuderi et al., 2013

Rheumatoid Arthritis	ASCs (3D-spheroids intra-articular injection)	Suppression of proliferation and migration of activated inflammatory cells and downregulation of inflammatory cytokines; suppression of synovial cell and macrophage function, upregulation of TSG-6 and TGFβ1	El-Jawhari et al., 2014; Ueyama et al., 2020

Systemic Lupus Erythematosus	ASCs (intravenous injection)	Immunomodulatory effects: decreased serum levels of immunoglobulins (IgG, IgG1, IgM, and IgE) and autoantibodies; decreased number of Th1 cells and Th17 cells; increased Foxp3- expressing Tregs, which suppress autoimmune processes and maintain peripheral tolerance	Park et al., 2015

Type I Diabetes	ASCs (intra-peritoneal injection)	Recovered levels of glucose, cholesterol, triglycerides, urea nitrogen, and creatinine; alleviation of renal damage through reduction of oxidative stress; decreased TNF-α, IL-1β, and IL-6 cytokine levels and inhibition of the MAPK signalling pathway; improved pancreatic expression of insulin and pancreatic duodenal homeobox 1 (Pdx1); attenuation of Th1 immune response	Vanikar et al., 2010; Lin et al., 2015

Inflammatory Bowel Disease (e.g. Crohn’s disease)	ASCs (local application and systemic infusion)	Anti-inflammatory effect, down-regulation of Th1-type cytokines expression, IFN-γ and TNF-α, increase of the number of CD4 T cells producing IL-10; proliferation, angiogenesis and remodeling stimulation during the healing process	González et al., 2009a; Panés et al., 2016; Sovrea et al., 2019

GvHD	ASCs (systemic infusion)	Prevention of allogeneic T-cells proliferation; suppression of the proliferation of T cells induced either by mitogens or allogeneic cells; secretion of soluble factors with immuno-suppressive effects; inhibition of inflammatory cytokines production (TNF-α, IFN-γ, and IL-12) of stimulated T cells	Yañez et al., 2006; Fang et al., 2007; Tholpady et al., 2009

Despite the widespread use of ASCs in cell therapy trials, the pathophysiological mechanisms at the basis of their clinical use have been not yet fully investigated. Here, we focused on the immunomodulatory properties of ASCs, through a comprehensive description of the molecular mechanisms and factors involved and the importance of milieu chemical-physical characteristics. Indeed, a plethora of paracrine signals are involved in ASC-mediated immunomodulation, thus suggesting the potential clinical use of ASC secretome, and the importance of environmental stimuli (culture conditions as well as local *in vivo* microenvironment) in determining clinical outcome. We also shed light on the potential strategies for optimization of ASCs efficacy in clinical settings, especially those related to boosting immunomodulatory capacity, acting both on local microenvironment and directly on ASCs through genetic or epigenetic regulation of gene expression, and to improving ASC characterization in order to standardize clinical outcome.

## Immunomodulatory Properties of ASCs

ASCs are considered more powerful suppressors of immune response than mesenchymal stem cells (MSCs) derived from different tissue sources, including trabecular bone, bone marrow, dental pulp, and umbilical cord (Ribeiro et al., 2013; Nancarrow-Lei et al., 2017). In particular, ASCs immunomodulatory effects exceeds that of bone marrow MSCs, which are currently considered the gold standard, due to a higher level of cytokines secretion (Melief et al., 2013). Indeed, a small number of highly metabolically active ASCs secrete higher amount of immune suppressive cytokines, such as IL-6 and transforming growth factor-β1 (TGF-β1) (Soleymaninejadian et al., 2012; Melief et al., 2013; Montespan et al., 2014). In the allogeneic setting, where HLA mismatch occurs, such as in mixed lymphocyte reactions (MLR), ASCs suppress T cell allo-proliferation, because they express low levels of MHC-I, are deficient in major MHC-II and in costimulatory molecules, like CD80, CD86, CD40 and its ligand, CD40L (Strioga et al., 2012; Machado et al., 2013). ASCs also inhibit B lymphocyte proliferation and differentiation into plasmocytic cells, unable to produce antibodies (Franquesa et al., 2015). This characteristic could be important for their use in the treatment of B-cell mediated disorders and GvHD during organ transplantation (Franquesa et al., 2012, 2015). B-cells are antigen-presenting cells (APCs) that produce pro- and anti-inflammatory cytokines. When both ASCs and B-cells are in a co-culture setting, the former cells have an inhibitory effect on the chemotactic properties of the latter, by downregulating chemokine receptors on the B cells, such as CXCR4, CXCR5 (Corcione et al., 2006). In contrast, ASCs may induce proliferation of a subset of CD5 + regulatory B cells that secrete immunosuppressive IL-10. This cytokine inhibits the production of other inflammatory cytokines by activated T cells and could be relevant in the therapeutic treatment of autoimmune diseases (Kalampokis et al., 2013; Peng et al., 2015). In addition, a negative feedback loop between activated T cells-producing interferon γ (IFNγ) and ASCs exists (Machado et al., 2013). The secretion of IFNγ primes ASCs against T cells proliferation (Machado et al., 2013). Consequently, ASCs are able to escape immune surveillance and simultaneously, they are endowed with ability to self-renew and differentiate into other cell-like types, hence facilitating allogeneic tissue regeneration (Machado et al., 2013; Bateman et al., 2018). A relevant example of the immunomodulatory potential of allogeneic ASCs is the injection of human ASCs (hASCs) in a DBA/1 mouse model with collagen-induced arthritis (González et al., 2009b). Administration of hASCs provoked a decrease of several inflammatory cytokines and chemokines, resulting in reduction of antigen-specific Th1/Th17 cell proliferation. In contrast, IL-10 production was induced in lymph nodes and joints. Yet, antigen-specific Tregs were also produced and as a consequence, self-reactive T effector responses were suppressed (González et al., 2009b).

The stromal vascular fraction (SVF) of an adipose tissue contains not only ASCs but also different cell types, such as endothelial cells, pericytes, lymphocytes, monocytes macrophages, fibroblasts, and smooth muscle cells (Dominici et al., 2006; Bourin et al., 2013; Nürnberger et al., 2019). *In vitro*, freshly isolated SVF containing ASCs secrete trophic and pro-regenerative factors, such as cytokines, growth factors, anti-inflammatory factors and extracellular vesicles harboring proteins or even microRNAs, collectively known as secretome (Fu et al., 2017). In fact, several studies have shown that the paracrine effects of ASCs and not the cells themselves, are pivotal players for tissue repair along with angiogenic and immunomodulatory properties occurring at the site of the damaged tissue (Tögel et al., 2005; Krampera, 2011; Eleuteri and Fierabracci, 2019). For example, high levels of two immunomodulatory mediators, indoleamine-pyrrole 2,3-dioxygenase (IDO) and Prostaglandin E2 (PGE2), were detected at 24 h in the supernatants from freshly isolated SVF (Nürnberger et al., 2019). It is known that IDO is an immunomodulatory enzyme produced by macrophages with immunosuppressive functions for T-cells and natural killers (NK). Moreover, ASCs inhibit maturation of dendritic cells and induce macrophage to differentiate into anti-inflammatory regulatory cells (Kim and Hematti, 2009; Sun et al., 2019). PGE2 is a major mediator of ASCs immunomodulation having multiple functions (Gao et al., 2016; Najar et al., 2016; Kota et al., 2017). One of these is the induction of IL-10 by macrophages, which in turn inhibits NK cells and T helper cells (Eleuteri and Fierabracci, 2019). Human leukocyte antigen-G5 (HLA-G5), galectins and programmed cell death ligand (PD-L1) are other factors involved in immunomodulation by ASCs (Gieseke et al., 2010; Yang et al., 2012; Zhou et al., 2018). HLA-G5 is a non-classical MHC- class I molecule, expressed on the surface of ASCs, which exert immunogenic tolerance through inhibition of NK, allogeneic T-cell responses and dendritic cells (DC) (Nasef et al., 2007). Galectin-1 and PD-L1 are negative regulators of immune responses, known as immune checkpoints (ICs) and are expressed on the surface of ASCs (Najar et al., 2010; Zhou et al., 2018). A milestone study conducted by Sheng et al. (2008) showed that PD-L1 expression is induced on the surface of MSCs isolated from mouse bone marrow by T cell secreted IFNγ. This study provided further insights as to the role of PD-L1 and other ICs in the immunosuppressive potential of ASCs. Zhou et al. (2018) co-cultured human ASCs that express PD-L1 and galectin 9 (Gal-9), with allogeneic peripheral blood mononuclear cells (PBMCs). The two ICs bound the corresponding ligands on the T cell, PD-L1 to PD-1 and Gal-9 to TIMP-3. In these experimental conditions, ASCs evoked T cell suppression through inhibition of the transcription factor NF-κB activation in TCR-stimulated T cells via the PD-L1/PD-1 and Gal-9/TIMP-3 pathways (Zhou et al., 2018). However, the interaction between ICs and ASCs has not been explored fully and this could be an exciting area of further investigation.

The immunosuppressive ability of ASCs is both dose and cell passage dependent (Waldner et al., 2018). After the isolation of ASCs from adipose tissues, these cells undergo multiple culture passages *in vitro*, since significant numbers are needed for autologous clinical applications. Critically, during serial passaging, ASCs become more differentiated and simultaneously they start losing their immunosuppressive properties. For instance, HLA-G expression dramatically decreases in adult ASCs (Teklemariam et al., 2014). Furthermore, the expression of HLA class-II and the other co-stimulatory molecules, CD80 and CD86, increases in very late passaged ASCs differentiating in mature adipocytes then causing T-cell proliferation in a direct contact-dependent manner (Poloni et al., 2015). Changes in cytokine milieu chemical-physical characteristics with subsequent passages of ASCs, also need to be taken into consideration (Leto Barone et al., 2013). Therapeutic applications of ASCs are influenced by hypoxic conditions, 3D cell culture scaffolds and growth factors added in culture, that could modify cell proliferation and ultimately their secretome (Madrigal et al., 2014). Once the ASCs or their secretome are injected into an injured tissue, they might interact with an unfavorable microenvironment, provoking different therapeutic responses than expected. For this reason, it has been suggested to evaluate the inflammatory status and the disease stage of a patient prior to the recruitment and treatment for clinical trials (Eleuteri and Fierabracci, 2019). The safety and timing of ASCs administration are also important requirements for the favorable outcome of cell therapy (Leto Barone et al., 2013).

## Approaches for Optimization of ASCs Immunomodulatory Efficacy

Although ASCs demonstrated higher immunomodulatory potential with respect to other kind of MSCs (Ivanova-Todorova et al., 2009; Najar et al., 2010, 2013; Ribeiro et al., 2013), their use as a gold standard for cell therapy of immune-related disorders needs further elucidations. Once ASCs are isolated and expanded in 2D cultures *in vitro*, they lose the stem cell niche environmental protection and sustainment, which is important to maintain ASC pluripotency, but also their immunomodulatory properties (Jones and Wagers, 2008; Napoli et al., 2008; Voog and Jones, 2010; Kaewsuwan et al., 2012). To date, different ASC enhancement strategies have been proposed, based on external stimuli changes and interventions focused on cells themselves.

An intuitive solution could be to directly recapitulate stem cell niche setting through 3D cultures. In these multicellular structures, adherent cells aggregate with each other through suspension culture system, generating spheroids (Kapur et al., 2012; Daquinag et al., 2013; Duval et al., 2017). As such, paracrine signaling is promoted by the proximity of the cells and the interaction of ASCs with extra cellular matrix (ECM)-like structures or other cell types (co-cultures) increases the secretion level of anti-inflammatory and pro-angiogenic molecules (Amos et al., 2010; Bartosh et al., 2010; Mineda et al., 2015). The employment of ASCs spheroids in the treatment of experimental *in vivo* disease models such as elastase-induced emphysema, hindlimb ischemia, acute kidney ischemia and diabetic skin wound healing demonstrated encouraging results regarding differentiation, pro-angiogenic and regenerative capacity of ASCs (Amos et al., 2010; Xu et al., 2016; Cho et al., 2017; Park et al., 2017). Currently, chitosan-coated culture plates, concaved bottom wells, ultra-low attachment plates, the hanging drop technique and spinner flask are the most widely used methods for spheroid generation (Duval et al., 2017). In particular, Regmi et al. (2019) showed that 3D-ASCs obtained through hanging drop method have enhanced immunomodulatory effects in systemic inflammatory response syndrome frequently observed in severe fulminant hepatic failure. Limitations of 3D spheroids may be related to the fact that *in vitro* nutrients and oxygen may be less available at the core of the 3D spheres, inducing necrosis and altering ASCs functions (Cheng et al., 2013; Edmondson et al., 2014). An alternative strategy to obtain 3D ASCs cell cultures is simulate microgravity in a Rotary Cell Culture System (RCCS) bioreactor (Yu et al., 2011; Kang et al., 2015). The RCCS system can be useful to overcome the oxygen and nutrient gradient problems (Costantini et al., 2019). Anyway, in these experimental conditions, some other factors involved in immunomodulation could be harder to control. An easier strategy may be to dissect these mechanisms and apply single level interventions.

As for the external stimuli, it has been demonstrated that the presence of growth factors in the culture medium could enhance ASCs differentiation (Ceccarelli et al., 2018). Likewise, pre-conditioning of ASCs with cytokines or other bioactive molecules represents one of the main approaches to boost ASCs immune regulatory functions before their therapeutic administration. IFN-γ and tumor necrosis factor α (TNF-α) are the major inflammatory cytokines used in MSC functional enhancement. After IFN-γ priming, ASCs showed overexpression of IDO, together with Cyclooxygenase 2 (COX2), TGF-β and Hepatocyte Growth Factor (HGF) (Ryan et al., 2007; Delarosa et al., 2009; Kronsteiner et al., 2011), central factors in immunological tolerance and anti-inflammatory mechanisms. TNF-α treatment, not only increased the production of IDO, PGE2, and HGF, but also of the pro-inflammatory cytokines IL-6 and IL-8, promoting endothelial progenitor cell migration and angiogenesis (Crop et al., 2010; Kwon et al., 2013), a primary goal of regenerative medicine. Furthermore, ASCs primed with IL-17, TNF-α, and IFN-γ have increased T cell immunosuppressive capacity mediated by inducible nitric oxide synthase (iNOS) production and have been shown to reduce inflammation and tissue injury in murine model of hepatitis (Han et al., 2014). An additional strategy is the pre-conditioning of ASCs with toll-like receptors (TLRs) agonists. ASCs express TLRs, cell membrane sensors that play a pivotal role in innate immune system mechanisms (Hwa Cho et al., 2006; Lombardo et al., 2009). Although TLRs agonists seem to be mainly implicated in ASCs multi-lineage differentiation capacity (Seo et al., 2019), studies demonstrated that TLR3 activation might induce suppressive phenotype (Hwa Cho et al., 2006; Bunnell et al., 2010; Waterman et al., 2010). By mimicking an *in vivo* inflammatory milieu, the combination of cytokines and TLRs ligands seemed to generate addictive effects on MSCs immunomodulatory properties, enhancing their therapeutic efficacy more than the use of a single molecular category (Gu et al., 2015). The downside of this approach may be that cytokine priming can confer immunogenicity to the ASCs, exposing them to host immune responses (Galipeau, 2017).

To date, only few studies have been performed on the induction of epigenetic modification to enhance immunomodulatory capacity of ASCs. Recent findings have provided insight into the exposure to 5-Aza-2′-deoxycytidine (5-AZA-dC) during *in vitro* expansion of ASCs that resulted in the upregulation of HLA-G gene, whose sustained expression is crucial to maintain immunomodulatory capabilities in adult stem cells (Teklemariam et al., 2014). MicroRNAs are also implicated in epigenetic regulation of ASCs immunomodulatory properties, as proven by Wang et al. (2019) in a mouse model of colitis. Mysm1, a histone deubiquitinase, is induced by TNF-α and IFNγ in ASCs and promotes miR-150 transcription, which enhances iNOS production. Nitric oxide is catalyzed by iNOS that is essential for the immunosuppressive capacity of ASCs. Therefore, miRNA-based strategies could be promising in enhancing therapeutic ASCs efficacy. Because both epigenetic modulators and ASCs have common aspects in immune modulation (Lee et al., 2015; Sabia et al., 2017), their respective contributions should be established and it is important to pay attention to possible cross-interactions.

A straightforward approach is to modify MSCs in order to increase the expression of genes such as IL-10 (Min et al., 2007), HGF (Bian et al., 2009), IDO (Kim et al., 2018), and FOXP3 (Qi et al., 2015) to obtain therapeutic anti-inflammatory effects. Genetically engineered ASCs have demonstrated efficacy in the treatment of inflammatory disease *in vivo*. Several studies reported the clinical success of different approaches to ASC genetic modification. The administration of human ASCs transduced with a bicistronic lentiviral vector encoding mouse IL-4 in C57Bl/6 mice with experimental autoimmune encephalomyelitis (EAE), a model of multiple sclerosis, results in a reduction of antigen-specific T-cell responses, thus attenuating clinical disease (Payne et al., 2012). Human ASCs transduced with a lentiviral vector for CTLA-4 Ig overexpression, when transplanted into a mouse model of sustained severe collagen induced arthritis, are able to enable CTLA-4 binding to CD28, which results in the induction of T-cell clonal anergy and the amelioration of autoimmune disease (Choi et al., 2016). The introduction of IL-1 receptor-like-1 (sST2), a decoy receptor for IL-33, in ASCs using a bicistronic lentiviral vector encoding the sST2–C-terminal promotes inflammation suppression and alleviation of the pathological events in acute lung injury (ALI) mouse model (Martínez-González et al., 2013). Furthermore, overexpression of IL-35, a recently discovered anti-inflammatory cytokine, represent a powerful tool to potentiate ASC-based cell therapy approach for auto-immune diseases. Results obtained by Zhao et al. demonstrated that murine ASCs transduced with a recombinant lentiviral vector to overexpress IL-35 are able to decrease CD4 + T-cell proliferation and IL-17 secretion in an *in vitro* co-culture model (Zhao et al., 2017).

## The Road Ahead in Immunomodulatory Potential of ASCs

Much effort has been done regarding the use of autologous versus allogeneic cells for *in vivo* applications (Feisst et al., 2015), with the first being preferred especially for chronic pathologies since the time required for the isolation and expansion of cells is not a limit given to the non-acute nature of the diseases. However, by using autologous ASCs there were consistent variations in clinical outcome, since cell characteristics may vary between patients, not only according to biological factors as age, sex, body mass index but also depending upon the disease (Varghese et al., 2017).

In this regard, contradictory results are available about the properties of ASCs isolated from patients affected by autoimmune and chronic inflammatory diseases. For example, some reports demonstrated that ASCs from patients affected by systemic sclerosis show the same phenotypical and functional characteristics of their healthy counterparts (Scuderi et al., 2013; Capelli et al., 2017; Velier et al., 2019), while Griffin et al. (2017) observed that ASCs from sclerodermic patients showed identical phenotype and differentiation capacity of those from healthy donors, but displayed reduced proliferation and migration capacity. Whilst, the presence of cardiovascular risk factors in cardiac patients seems to reduce ASC pluripotency and self-renewal, thus discouraging their autologous use in the clinical setting (Dimmeler and Leri, 2008; Krawiec et al., 2016). In particular, microenvironmental factors and metabolic disorders may impact the functionality of these cells. ASCs derived from obese subjects and from patients affected by type 2 diabetes showed increased expression of inflammatory markers with respect to those derived from lean donors, as well as a remarkable reduction in their immunosuppressive activities (Serena et al., 2016). Moreover, ASCs from obese patients also display an impaired angiogenic potential (Oñate et al., 2012, 2013). Other groups observed that diabetes did not alter ASC isolation efficiency, growth curves and angiogenic potential, but ASCs from diabetic patients showed a delay in the acquisition of endothelial cell markers, thus suggesting an impaired differentiation (Policha et al., 2014).

The use of allogeneic stem cells may overcome these limitations, but it has to be considered that adipose tissue availability is dependent on surgical procedures, thus limiting the occurrence of overall healthy donors. More attention should be also paid to the origin of allogeneic ASCs, since several studies underlined a cellular and molecular variability depending on the donor age, sex and tissue source even under similar genetic and environmental conditions (Shu et al., 2012; Bodle et al., 2014; Ock et al., 2016; Abbo et al., 2017). In particular, ASCs derived from different donors might have significant variations in the chemical-physical characteristics of their secretome (Alicka et al., 2019), thus affecting their immunomodulatory capacity (Bunnell et al., 2010). In order to avoid donor-to-donor heterogeneity, a strategy with pooled ASCs of different allogeneic donors could be proposed, as previously reported for BMSCs (Kuçi et al., 2016), but immunogenic stimuli could increase (Ankrum et al., 2014; Patrikoski et al., 2014). Indeed, although ASCs have been shown to possess a low immunogenic profile, the potential immunogenicity of allogeneic cells, which might determine their rejection after infusion, cannot be excluded. In fact, it has been observed that the immunogenicity of ASCs decreases with cell passaging, so that cells at low passages are more immunogenic than those at higher passages (McIntosh et al., 2006), and that ASCs are not fully immune privileged, since they elicit both humoral and cellular immune response *in vivo*, depending on the microenvironment (Ankrum et al., 2014). Also ASC differentiation may alter their immunogenic phenotype, increasing HLA class-I and HLA class-II expression (García-Sancho et al., 2017), as well as culturing condition (e.g., use of human serum or serum-free conditions) (Patrikoski et al., 2014).

## Conclusion

ASCs represent a valuable treatment option for a wide range of inflammatory or autoimmune diseases, which therapeutic efficacy relies primarily on immunomodulatory activities mediate by paracrine effects. An increasing number of preclinical studies and clinical trials are being developed to assess ASCs safety and efficacy. However, although there are promising results and increasing knowledge in the *in vivo* applications of ASCs, unfortunately, several clinical trials have failed due to differences in experimental protocols, read-out, animal models and variability in ASCs characteristics. So, the clinical translation of ASCs still requires a proper validation in large controlled trials. For a more successful outcome of ASC based therapies, thorough investigations with more standardized protocols are urgently required, as well as a better understand of ASC immunomodulatory network and the identification of key molecules and/or regulatory mechanisms responsible for ASC effects in chronic inflammatory diseases.

**FIGURE 1:**
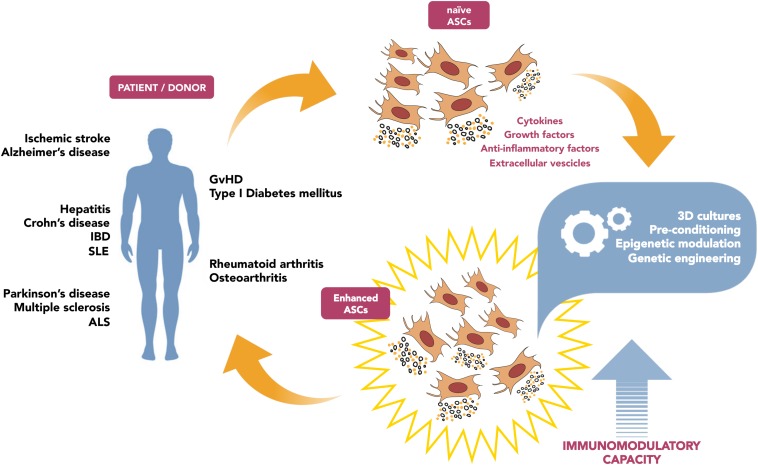
Schematic diagram illustrating the potential strategies aimed to boost ASC-mediated immunomodulation, in order to improve clinical outcome.

The in-depth analysis of the potential strategies aimed to boost ASC-mediated immunomodulation (summarized in Figure 1) will foster new targeted approaches for cell therapy applications in the field of immune diseases. Yet, such interventions need to be accurately considered, since any alteration of immune system sensitivity can be dangerous: weakening the capacity of recognize transformed cells or pathogens, or vice versa exacerbating immune response, could lead to adverse events. As for genetic modification, the engineered cells persisting in the host could generate undesirable effects, such as potential tumorigenicity related to genetic instability (Heslop et al., 2015; Neri, 2019).

Finally, standardization of both ASC and patient’s characteristics prior to clinical use are necessary to avoid that the donor phenotype might compromise their immunomodulatory properties, thus impairing their therapeutic efficacy.

## Author Contributions

SC conceived the review and wrote the manuscript. PP and EA revised the literature and helped to writing the manuscript. CN edited the manuscript. CM supervised the overall project and edited the manuscript. All authors had the opportunity to discuss and comment on the manuscript.

## Conflict of Interest

The authors declare that the research was conducted in the absence of any commercial or financial relationships that could be construed as a potential conflict of interest.

## Footnotes

1 www.clinicaltrials.gov
